# *Anadara granosa* shell powder improves the metabolism, testosterone level, and sound frequency of Pelung chickens

**DOI:** 10.14202/vetworld.2021.1564-1571

**Published:** 2021-06-18

**Authors:** Rizki Fitrawan Yuneldi, Pudji Astuti, Hendry T. S. Saragih, Claude Mona Airin

**Affiliations:** 1 Veterinary Science Postgraduate Study Program, Faculty of Veterinary Medicine, Universitas Gadjah Mada, Yogyakarta, Indonesia; 2 Department of Physiology, Faculty of Veterinary Medicine, Universitas Gadjah Mada, Yogyakarta, Indonesia; 3 Laboratory of Animal Development Structure, Faculty of Biology, Universitas Gadjah Mada, Yogyakarta, Indonesia

**Keywords:** natural aromatase blocker, Pelung, sound, testosterone, thyroid hormone

## Abstract

**Background and Aim:**

Pelung chickens make unique, pleasant, and rhythmic sounds in addition to having strong muscle mass. Performance is controlled by testosterone. A natural aromatase blocker is an agent capable of blocking the aromatase enzyme, which consequently prevents testosterone from being changed into estradiol. Such a condition results in consistently high testosterone levels. The aim of this study was to determine the effect of the administration of the shell powder of *Anadara granosa* on the testosterone level, frequency and quality of sounds, and metabolism of Pelung chickens within set parameters of thyroid hormone levels, the triiodothyronine (T_3_)/thyroxine (T_4_) ratio, and weight gain.

**Materials and Methods:**

This study used four Pelung chickens aged 14 months. Control data consisted of data before treatment (day 0). Shell powder of *A. granosa* was administered for 56 days. Body weight (BW) was measured, and blood samples were drawn. In addition, the sounds from Pelung chickens were recorded once every 2 weeks on days 14, 28, 42, and 56. The blood samples were analyzed using the enzyme immunoassay method to determine testosterone, T_3_, and T_4_ levels. The sounds of Pelung chickens were recorded using the Hagemon touch method and analyzed using the Audacity application.

**Results:**

The results showed a significant difference (p<0.05) in the levels of testosterone, BW, and sound frequency after the administration of *A. granosa* shell powder, but the administration did not have a significant effect on the levels of T_4_ and T_3_ hormones (thyroid hormone), T_3_/T_4_ ratio, or sound duration. The testosterone content and BW of Pelung chickens increased from day 14 to day 56, whereas T_3_ was indicative of the same profile as the T_4_ hormone. However, T_3_ and T_4_ levels and the T_3_/T_4_ ratio decreased and increased, respectively. The sound frequency of Pelung chickens increased from day 0 to day 14, although sound duration decreased.

**Conclusion:**

The administration of *A. granosa* shell powder at 0.9 mg/kg BW orally could improve the metabolism, testosterone level, BW, and sound frequency of Pelung chickens.

## Introduction

Pelung chickens are endemic to Cianjur, Indonesia. The chickens are well known abroad and have high economic value. Decree Nomor 2918/kpts/OT.140/6/2011 issued by the Ministry of Agriculture officially [1] declared Pelung as an indigenous chicken breed of Indonesia that requires protection and conservation. Pelung chickens are threatened because of their reduced population sizes [2]. Pelung chickens have a distinctive, pleasant, long, rhythmic crow, and it has been shown that the longer and the more melodious the sound, the better Pelung chickens perform [2,3]. Another characteristic of the chicken is the increased mass and strength of their muscles [4]. The performance of Pelung chickens is controlled by testosterone, and good performance is highly important in improving the quality of the cocks. The aromatase enzyme can change the testosterone in chickens into estradiol, and consequently, high testosterone levels cannot be maintained [5].

At present, Pelung chickens grow relatively fast and show good performance, with the males cross-breeding with other free-range chickens, thereby improving the genetics of the chickens. According to Sopian *et al*. [6], the cross-breeding of Pelung chickens that have a far kinship with other free-range chickens and free-range chickens will theoretically provide the offspring with better performance. The thyroid gland produces thyroxine (T_4_) and triiodothyronine (T_3_) hormones. According to Mullur *et al*. [7], the thyroid hormone serves an important function in basal metabolic regulation and thermogenesis stimulation, whereas under normal conditions, the hormone can control metabolic rate and energy balance. The decrease in thyroid activity correlates to the decrease in metabolic regulation. A total of 99% of the thyroid hormone circulating in blood is bound to plasma proteins, such as globulin and transthyretin. The thyroid and testosterone hormones are the most significant in interaction with zinc (Zn). Low Zn values in hypothyroidism and high Zn value in hyperthyroidism are indicative of the correlation between the Zn and the thyroid hormone [8]. Dittrich *et al*. [9] showed that the induction of testosterone in female robins correlated to the cellular differentiation process in the high vocal center. Direct injection of testosterone can prevent a decrease in testosterone levels. However, the direct injection of testosterone results in a downregulation mechanism, which causes a drastic decrease in testosterone content [10]. The administration of synthetic aromatase blocker results in the same outcome. The administration of aromatase blocker letrozole (lz; 1.5 mg/kg) can increase testosterone content and improve the reproduction performance of cocks after their peak age [11]. The supplementation of Zn at doses of 2.5-3 mg/kg/day for 6 weeks and weight exercise 4 times a week can significantly increase free and total testosterone content and positively impact human physical performance [5]. The administration of aromatase blocker Taxadrol can increase testosterone content [12].

Shells of *Anadara granosa* are usually disposed off and not used for any purpose [13]. According to Astuti *et al*. [14], the powder from shells of *A. granosa* contains Zn, magnesium (Mg), calcium, sodium (Na), iron (Fe), and potassium (K). The powder from the shells *A. granosa* also contains copper (Cu) and selenium (Se) [15]. Sahin *et al*. [16] suggested that a combination of Mg, Zn, and Se is more effective than the individual minerals in increasing testosterone levels and muscle mass. According to Astuti *et al*. [14], the Zn contained in the powder of *A. granosa* shells could play an important role as an aromatase blocker, which subsequently results in the absence of changes of testosterone into estrogen, such that blood testosterone content is consistently high. Some studies have shown that Zn, Mg, and vitamins in shellfish can increase testosterone content [5]. Supplementation with Zn can increase testosterone content in male rats [17]. It serves the function of an aromatase blocker that can decrease testosterone synthesis into estrogen [14,18]. Some studies have shown that Zn plays an important role in DNA synthesis, cell proliferation, immunocompetence, and maintaining nucleic and membrane integrity in which chromatin is considered to have the highest Zn concentration [19]. Consequently, it is necessary to determine methods to improve the performance of Pelung chickens.

The aim of this study was to determine the effects of *A. granosa* shell powder on body weight (BW), frequency and quality of sounds, thyroid hormone content representing one of the metabolic indicators, and testosterone hormone levels as an indicator of the performance of Pelung chickens.

## Materials and Methods

### Ethical approval

The study protocol was approved by Integrated Testing and Research, Universitas Gadjah Mada (UGM), (00020/04/LPPT/V/2020).

### Study period and location

The study was conducted from April to December 2020 in the Department of Physiology, Faculty of Veterinary Medicine, Universitas Gadjah Mada, and the poultry farming in Bantul, Yogyakarta, Indonesia.

### Preparation of *A. granosa* shell powder

*A. granosa* is sourced from the north coast of Semarang and identified by Head of the Animal Systematics Laboratory, Faculty of Biology, UGM, namely, Dr. Dra. Rr. Upiek Ngesti W. Astuti, DAP., M.Kes.

*A. granosa* shell powder was prepared by first boiling the shells and then separating the meat from the shells, and subsequently, the shells were cleaned and dried under sunlight for 1-2 days. Next, the dried shells were boiled in NaOH (1.0 N) solution at 50°C for 3 h and rinsed in flowing water. Subsequently, the shells were dried using an oven at 120°C for 6-8 h. Once they were dry, they were pressed into powder [14].

### Animals and treatment

The study used four male Pelung cocks at 14 months of age to observe the profiles of T_3_, T_4_, testosterone, BW, sound frequency, and sound quality. Pelung chickens were analyzed for 7 days. Blood samples were drawn before administering *A. granosa* shell powder (day 0). Day 8 was considered the 1^st^ day shell powder was mixed into feed, which continued for 48 days at a dose of 0.9 mg/kg BW, as modified by Astuti *et al*. [14]. The blood sampling and voice recording were conducted once every 2 weeks on days 14, 28, 42, and 56.

### Voice acoustic analysis

The sound of Pelung chickens was recorded once every 2 weeks using the Hagemon touch method for 60 min from 7:00 to 8:00 West Indonesia time [20]. The data obtained were analyzed using the Audacity application to determine the sound frequency and quality of Pelung chickens [2]. The voice quantity test was based on the number of voices every 10 min (frequency), whereas the quality of the voice was based on the duration of the sound of Pelung chickens [20].

### Testosterone analysis

#### Preparation of the wash buffer

The wash buffer used in the study was created by 20× liquefaction of 25 mL wash buffer solution and 475 mL distilled or deionized water. The resulting solution was kept at the temperature of 18-26°C before it was used.

#### Analytical procedure

The analysis was conducted according to the manufacturer’s instructions in the KITs. First, 50 μL standard solution and samples were poured into wells, and 100 μL enzyme conjugate reagent was added and incubated for 60 min. Next, the wells were rinsed 3 times using 300 μL wash buffer. Subsequently, 100-300 μL TMB substrate reagent was added to the wells, and the microplate was covered and incubated for 30 min. Then, 50 μL stop solution was added to the wells, and absorbance was read using an ELISA or microplate reader (ZENIX-320) at the absorbance of 450 nm for 15 min.

### T_3_ and T_4_ analysis

#### Preparation of conjugate enzyme

The conjugate enzyme consisted of the T_4_ enzyme conjugate and assay diluent at the ratio of 1:11. The study used all of the plates such that there were 700 microns T_4_ enzyme conjugate in 0.7 mL assay diluents. Once the conjugate solution has been prepared, it was not stored for more than 24 h.

#### Analytical procedure

The analysis was conducted according to the manufacturer’s instructions in the KITs. First, 50 mL of the standard solution and sample was poured into wells, and 100 μL enzyme conjugate reagent was added. Next, 50 μL biotin reagent was poured into the wells and incubated for 30 min. Subsequently, the wells were rinsed 3 times using 300 μL deionized water. Then, 100 μL TMB substrate reagent was added to the wells, and the microplates were covered and incubated for 30 min. Finally, 50 μL of stop solution was added to the wells, and absorbance was read using ELISA or microplate reader (ZENIX-320) at the absorbance of 450 nm for 15 min.

### Statistical analysis

The results of the readings from the ELISA reader were in the form of optical density (OD) and were processed using Microsoft Excel software such that they could be applied to testosterone concentration (ng/mL) [21]. The testosterone, T_3_, T_4,_ BW, sound frequency, and sound quality values were statistically analyzed using an analysis of variance at the confidence level of 95% (a = 0.05). If there was a significant difference among them, the Duncan test was then conducted for pairwise comparisons. The analysis was conducted using SPSS software, version 15 [22].

## Results

### Thyroid hormone

**Figure-1:**
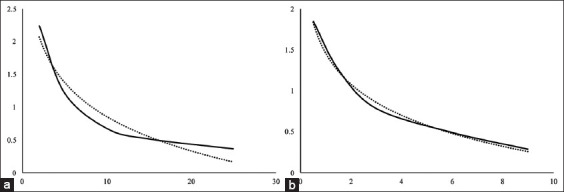
Standard curve of the hormones (a) triiodothyronine T3, R²=0.9439 and (b) thyroxine, R²=0.9955.

The T_3_ and T_4_ contents were examined using a competitive EIA method capable of converting the OD value into content with a standard curve and the formulas y = –0.756ln(x) + 2.5957 for the T_3_ standard and y = –0.546ln(x) + 1.457 for the T_4_ standard. The standard curve of the two hormones is presented in Figure-1. The results showed that the peroral administration of *A. granosa* shell powder at the dose of 0.9 mg/kg BW could improve metabolism and significantly impact the increase in the secretion of testosterone (Table-1). In addition, there was no significant increase or decrease in the T_3_ and T_4_ contents such that the T_3_/T_4_ ratio was stable.

**Table-1:** Data from the measurements of testosterone, T_3_, T_4_, and body weight of Pelung cocks.

Parameters	Average±SD, days				

	0	14	28	42	56
Level of T_3_ (ng/dL)	0.50±0.46	0.55±0.39	0.42±0.36	0.47±0.29	0.46±0.30
Level of T_4_ (mcg/mL)	3.96±0.87	3.21±1.29	2.94±0.96	3.29±1.65	3.33±1.67
Ratio of T_3_/T_4_	0.13±0.08	0.14±0.06	0.16±0.16	0.15±0.13	0.18±0.16
Level of testosterone (ng/mL)	0.13±0.06^e^	0.35±0.04^d^	1.10±0.05^c^	1.27±0.04^b^	1.42±0.06^a^
Body weight (kg)	2.70±0.14^c^	3.75±0.54^b^	3.82±0.55^ab^	4.03±0.51^ab^	4.52±0.40^a^

^a-e^Different superscripts in the same row indicate significant differences (p<0.05). T_3_=Triiodothyronine, T_4_=Thyroxine

**Figure-2:**
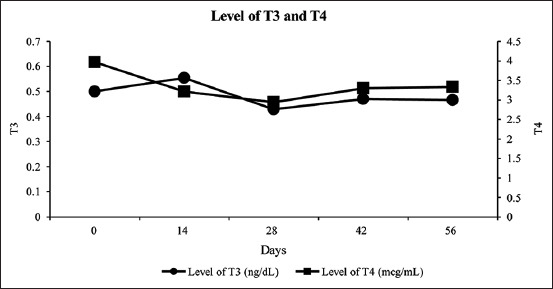
Triiodothyronine and thyroxine hormone levels in Pelung cocks before and after treatment (on the days 14, 28, 42, and 56).

The results showed no significant decrease in T_4_ followed by an increase in T_3_ (Figure-2). The profile of T_3_ and T_4_ in the study was constant. This indicated no significant increase in the thyroid hormone or any change of T_4_ into T_3_ in response to an imbalance in physiological changes.

### T_3_/T_4_ ratio and BW

**Figure-3:**
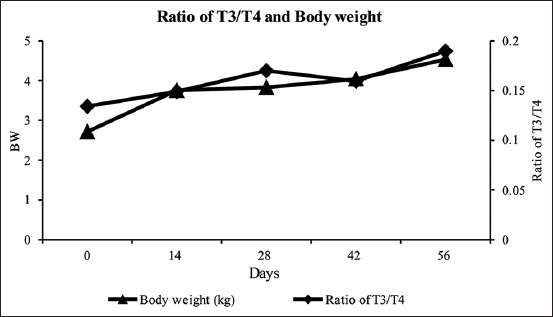
Triiodothyronine/thyroxine ratio and the body weight of Pelung chickens during treatment.

Metabolic abnormalities can be detected from the T_3_/T_4_ ratio. The results showed that the weekly T_3_/T_4_ ratio did not differ significantly (Table-1). As is the case in mammals, the increase in the T_3_/T_4_ ratio in chickens would significantly affect the decrease in the secretion of other hormones. The decrease in estradiol was influenced by low T_3_ content. It was expected that this happened because of the decrease in the CYP19 mRNA gene expression in granulosa cells [23]. In addition, the BW of Pelung chickens significantly increased at each measurement. The BW of Pelung chickens continuously increased from day 14 to day 56, as summarized in Table-1 and illustrated in Figure-3.

### Frequency and duration of Pelung chicken sounds

**Figure-4:**
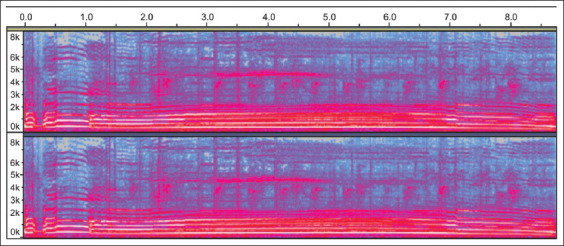
Pelung chicken crowing spectrogram.

The experiment used 14-month-old chickens that could be classified as adult chickens. According to Asmara *et al*. [2], the duration of the crow of Pelung cocks aged more than 12 months was 6.30-12.80 s, its frequency was 4-67 Hz, and its intensity (volume) reached 45.20-65.20 dB. The administration of *A. granosa* shell powder for 56 days decreased the sound duration but not significantly. The duration of sound in the experiment was considered normal (Table-2 and Figure-4).

**Table-2:** Data on the duration and frequency of Pelung chicken sounds during this experiment.

Sound	Days				

	0	14	28	42	56
Duration(s)					
Average±SD	7.53±3.09	5.77±3.00	5.62±2.98	5.16±2.95	4.97±3.04
Max	10.69	9.93	9.70	10.41	9.47
Min	3.27	3.11	2.92	3.09	2.88
Frequency (Hz)					
Average±SD	5.74±1.79^b^	12.48±1.38^a^	10.91±1.95^a^	11.53±2.53^a^	6.41±1.87^b^
Max	7.94	14.18	13.31	14.63	8.71
Min	3.53	10.78	8.51	8.45	4.11

^a,b^Different superscripts in the same row indicate significant differences (p<0.05)

The frequency of the crows of Pelung chickens in the experiment began to increase on days 14, 28, and 42 after the administration of the shell powder. The results of the experiment showed that there was a significant difference (p<0.05) in the frequency of the sound of Pelung chickens before treatment (days 0) with that after the administration of *A. granosa* shell powder (days 14, 28, 42), as shown in Table-2 and Figure-4.

### Testosterone hormone

**Figure-5:**
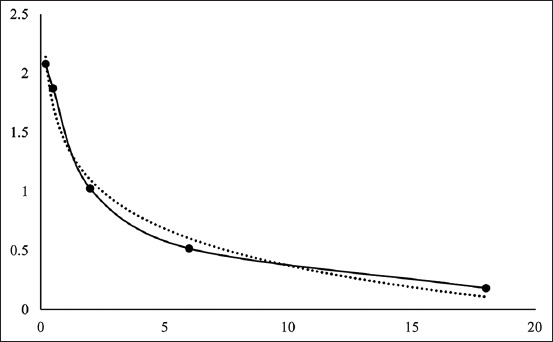
The standard curve of testosterone hormone, R²=0.9839.

**Figure-6:**
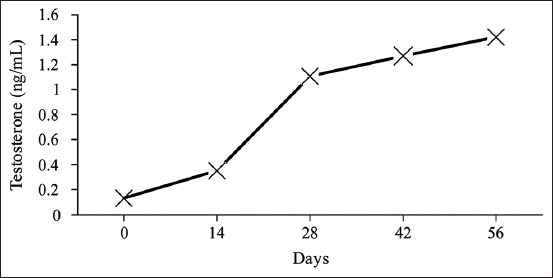
Testosterone content of Pelung chickens before (day 0) and after treatment (days 14, 28, 42, and 56).

Testosterone values resulted from the conversion of OD using a standard curve with the formula y = –0.451ln(x) + 1.4108, as illustrated in Figure-5. As shown in Table-1 and Figure-6, there were significant differences (p<0.05) in testosterone levels after administering *A. granosa* shell powder. The testosterone content continuously increased on days 14, 28, 42, and 56 (after treatment). This indicated that the Zn content of the shell powder increased the testosterone level in Pelung chickens.

## Discussion

### The effects of *A. granosa* shell powder on thyroid hormones

The administration of *A. granosa* shell powder could improve metabolism. According to Cunningham and Klein [24], normal metabolism is indicated by the absence of a decrease in the levels of T_4_ hormone, which would significantly affect the increase in the T_3_ hormone. The decrease in the T_4_ hormone indicates that there was a change in the T_4_ hormone into the T_3_ hormone. This might result from the increase in the conversion of T_4_ into T_3_ as an active hormone. The decrease in the T_4_ content is followed by an increase in T_3_ content. T_3_ was more active than the T_4_ hormone because the T_4_ hormone serves the function of a reserve before it is changed into T_3_ [25]. T_3_ regulates the development and the physiological aspects at the cellular level, including metabolism, proliferation, differentiation, and cell apoptosis [23].

The administration of *A. granosa* shell powder indirectly increased the intake of Zn. According to Kilic [8], low Zn levels occur with hypothyroidism, whereas a high Zn level is found with hyperthyroidism. The high concentration of Zn in the testes and accessory glands indicates that Zn plays an important role in the reproductive system [26]. This study showed that the administration of *A. granosa* shell powder, which contained Zn, helped optimize hormones, especially testosterone. This was consistent with the results of Khalil [27] who indicated that the supplement of Zn could increase the production of eggs and improve the efficiency of the use of ransom. According to Richards *et al*. [28], supplementation of Zn and Cu in chickens resulted in better stamina and health status of the digestive tract. The combination of Zn and Fe supported thyroid gland function [29].

### Effects of *A. granosa* shell powder on the T_3_/T_4_ ratio and BW

The development of Pelung chickens is faster than that of other chickens [30]. It is indicated by the BWs of 2.11 kg cocks at 20 weeks of age, 2.5 kg at 42 weeks of age, and 4 kg adults [31]. This study showed that the BW of Pelung chickens increased at each measurement. The statistical analysis showed that *A. granosa* shell powder treatment caused an increase in BW of Pelung chickens on day 56, and they were heavier than before the treatment. Compared to Pelung chickens with regular feed, the BW of Pelung chickens in this study was considered normal. It was assumed that the supplementation of Zn contained in the shell powder did not cause any physiological problems that could cause a decrease in the BW. According to Akhadiarto [32], the impact of the change in the feed formulation on the productivity of chickens could be observed in the existing production standard of chicken breeding, including BW, feed consumption, BW gain, and feed conversion. Iskandar and Susanti [31] suggested that if metabolism was good, it would increase BW, neck length, body length, beak width, the width of the fleshy part of the tail, and the position of the larynx from the lower jaw, which should be positively correlated to crow volume. In addition, the width of the neck muscle at the larynx, width of the neck muscle, and BW are positively correlated to the typical crow of Pelung chickens.

### Effects of *A. granosa* shell powder on the frequency and duration of sounds

Pelung chickens have different individual characteristics. A decrease in sound duration (sound quality) of Pelung chicken crow, although still within the normal range and not significantly different, might have been caused by the raising system during treatment. These findings are similar to those of Rusfidra [33], where Pelung chicken cooping system could be one of the causal factors of a decrease in sound quality. There were two causal factors of the decrease in the quality of the crow of Pelung cocks. First, the cocks were not allowed to mate, and second, it is thought that the presence of elder male cocks is important for mating [33]. There are probably other factors that influence sound duration, including internal (genetic) and external (nutrition and environmental) factors [34]. The profile of sound of Pelung chicken at 12 months of age or less, on average, has a total duration of 8.85 s, and the profile of the crow of Pelung chicken at 12 months of age or more has an average duration of 9.00 s [2]. Asmara *et al*. [3] suggested that the duration of the crow of Pelung chickens was in the range of 5.17-12.33 s.

The increase in sound frequency was probably caused by the administration of *A. granosa* shell powder as a natural aromatase blocker, which could block the aromatase enzyme, thereby increasing the levels of testosterone. Testosterone is an underlying factor for crowing and social dominance [35,36]. The chirp of the male birds is controlled by testosterone produced in both the Leydig cells and the brain [37]. These findings are similar to those of Astuti *et al*. [20] who showed that aromatase blockers could increase the male canary song frequency.

### Effects of *A. granosa* shell powder on testosterone levels

The Zn content of the powder could play an important role as an aromatase blocker, which can change testosterone into estrogen [14,38,39]. According to Park *et al*. [40], Zn represented an important nutrient in animal metabolism. Akram *et al*. [41] stated that Zn is an important nutrient because it is involved in various metabolic pathways. Zn in birds serves as an important nutrient and can be a food supplement to manipulate the chicken reproductive system. Omu *et al*. [19] showed that a Zn deficiency could result in a change in the function and weight of the testes because it could cause 40-70% decrease in germ cells in the total volume of the testes. Zn plays an important role in the reproductive organs. If Zn deficiency occurred, atrophy of seminiferous tubules and spermatogenesis disorder would follow [26]. Some studies showed that the increase in the testosterone content was consistent with good quality voice, strong muscles, and good comb growth [8,42-44]. In addition, the increase in testosterone was also consistent with the reproduction capability, as indicated by mating success [45].

It has been shown that the Zn content of *A. granosa* shell powder can increase the reproductive performance of Pelung chicken through the increase in testosterone. Zn plays an important role in the function of more than 300 enzymes. Some enzymes in the body depend on Zn availability, such as RNA polymerase, alcohol dehydrogenase, and carbonic anhydrase. In addition, alkaline phosphatase depends on Zn as a cofactor. Zn also plays an important role in forming active biological molecular structures, such as Cu-Zn dismutase, and influences apoptosis protein kinase C activities [19]. Zn is necessary for the growth and maintenance of body physiology in birds, including bone and feather growth, enzymes, and appetite regulation [40,46]. Optimal development requires a Zn percentage of approximately 0.0012-0.0018% [47]. The high concentration of Zn in the testes and accessory glands indicated that Zn plays an important role in the reproductive organs. A Zn deficiency could result in hypogonadism. Therefore, it can be concluded that there is a positive correlation between Zn and testosterone [19,26]. A deficiency in Zn during the development of the testes could affect testicular steroidogenesis. Omu *et al*. [19] showed that a decrease in testosterone content was observed in the group with Zn deficiency, which caused the apoptosis of Leydig cells. It has been shown that Zn is involved in the regulation of testosterone production. Testosterone is produced by Leydig cells, and it is an important hormone in spermatogenesis. Therefore, a deficiency of Zn could be related to cell apoptosis at the early stage of spermatogenesis and spermatocyte, which could cause hypogonadism.

*A. granosa* shell powder administration in adult chickens has been proven to increase testosterone and sound frequency, but it could not increase duration. Thus, it can be concluded that the administration of *A. granosa* shell powder would not improve the quality of the crow in the cocks that have already developed a good quality crow.

## Conclusion

The administration of 0.9 mg/kg BW *A. granosa* shell powder could improve the metabolism, testosterone level, BW, and sound frequency of Pelung chickens.

## Authors’ Contributions

PA, CMA, HTSS, and RFY planned the study and designed the experiment. The study was managed and supervised by PA and CMA. PA, CMA, and RFY recorded and analyzed samples. CMA and RFY drafted the manuscript. RFY revised the manuscript under the supervision of PA, CMA, and HTSS. All authors read and approved the final manuscript.
